# Critical involvement of ZEB2 in collagen fibrillogenesis: the molecular similarity between Mowat-Wilson syndrome and Ehlers-Danlos syndrome

**DOI:** 10.1038/srep46565

**Published:** 2017-04-19

**Authors:** Mika Teraishi, Mikiro Takaishi, Kimiko Nakajima, Mitsunori Ikeda, Yujiro Higashi, Shinji Shimoda, Yoshinobu Asada, Atsushi Hijikata, Osamu Ohara, Yoko Hiraki, Seiji Mizuno, Toshiyuki Fukada, Takahisa Furukawa, Nobuaki Wakamatsu, Shigetoshi Sano

**Affiliations:** 1 Department of Dermatology, Kochi Medical School, Nankoku 783-8505, Japan; 2 Department of Perinatology, Institute for Developmental Research, Aichi Human Service Center, Kasugai 480-0392, Japan; 3 Department of Anatomy, Tsurumi University School of Dental Medicine, Yokohama 230-8501, Japan; 4 Department of Pediatric Dentistry, Tsurumi University School of Dental Medicine, Yokohama 230-8501, Japan; 5 Department of Bioscience, Nagahama Institute of Bio-Science and Technology, Nagahama 526-0829, Japan; 6 Laboratory for Integrative Genomics, RIKEN IMS, Yokohama 230-0045, Japan; 7 Hiroshima Municipal Center for Child Health and Development, Hiroshima 732-0052, Japan; 8 Department of Pediatrics, Central Hospital, Aichi Human Service Center, Kasugai 480-0392, Japan; 9 Department of Molecular and Cellular Physiology, Faculty of Pharmaceutical Science, Tokushima Bunri University, Tokushima 770-8514, Japan; 10 Laboratory for Molecular and Developmental Biology, Institute for Protein Research, Osaka University, Suita 565-0871, Japan; 11 Department of Genetics, Institute for Developmental Research, Aichi Human Service Center, Kasugai 480-0392, Japan

## Abstract

Mowat-Wilson syndrome (MOWS) is a congenital disease caused by *de novo* heterozygous loss of function mutations or deletions of the *ZEB2* gene. MOWS patients show multiple anomalies including intellectual disability, a distinctive facial appearance, microcephaly, congenital heart defects and Hirschsprung disease. However, the skin manifestation(s) of patients with MOWS has not been documented in detail. Here, we recognized that MOWS patients exhibit many Ehlers-Danlos syndrome (EDS)-like symptoms, such as skin hyperextensibility, atrophic scars and joint hypermobility. MOWS patients showed a thinner dermal thickness and electron microscopy revealed miniaturized collagen fibrils. Notably, mice with a mesoderm-specific deletion of the *Zeb2* gene (*Zeb2*-cKO) demonstrated redundant skin, dermal hypoplasia and miniaturized collagen fibrils similar to those of MOWS patients. Dermal fibroblasts derived from *Zeb2*-cKO mice showed a decreased expression of extracellular matrix (ECM) molecules, such as collagens, whereas molecules involved in degradation of the ECM, such as matrix metalloproteinases (MMPs), were up-regulated. Furthermore, bleomycin-induced skin fibrosis was attenuated in *Zeb2*-cKO mice. We conclude that MOWS patients exhibit an EDS-like skin phenotype through alterations of collagen fibrillogenesis due to *ZEB2* mutations or deletions.

Mowat-Wilson syndrome (MOWS; OMIM 235730) is a congenital disease with the prevalence of 1 per 50,000–90,000 live births^1^,^2^,^3^. MOWS is characterized by a distinctive facial appearance, intellectual retardation, epilepsy and malformations including Hirschsprung disease, congenital heart defects and urogenital anomalies^4^,^5^. In 2001, the underlying cause of MOWS was identified as mutations or deletions in the *Zinc finger E-box-binding homeobox 2* gene (*ZEB2*, previously called *ZFHX1B* or *SIP1*) located at chromosome 2q22^6^,^7^. *ZEB2* encodes Smad Interacting Protein 1 (SIP1), which is a multidomain protein characterized by a central homeodomain, a CtBP-binding domain and a Smad-binding domain, as well as two separated, highly conserved zinc finger clusters at the N- and C-terminals^8^. Each zinc finger cluster can independently bind to CACCT(G) sequences in the promoter regions of genes involved in development and differentiation, including the E-cadherin promoter^9^, by which E-cadherin is down-regulated, leading to initiation of the epithelial-to-mesenchymal transition^10^. ZEB2 was originally identified as a transcriptional co-repressor for the transforming growth factor β (TGF-β) signaling pathway through binding to Smad proteins^8^,^11^. Since *ZEB2* is a highly evolutionarily conserved gene that is widely expressed during embryological development, mice that are homozygous deficient in the *Zeb2* gene have defects in neural crest cell migration, compromised development of the peripheral nervous system, craniofacial tissues and the heart, which results in lethality between E9.5 and E10.5^12^,^13^. Notably, neural crest-specific ablation of *Zeb2* caused craniofacial and gastrointestinal malformations that resemble symptoms in human patients with MOWS^14^. Patients with typical MOWS harbor deletions or truncating mutations of *ZEB2*, suggesting that haploinsufficiency is the pathogenic basis of MOWS, although no genotype-phenotype correlations have been suggested^2^.

Among various anomalies in multiple organs, skin symptoms in MOWS patients have not been frequently reported, if any. Some patients with MOWS were reported with fair complexion^15^ or raindrop depigmentation^16^. Here we report for the first time that most MOWS patients exhibit skin manifestations similar to those of patients with Ehlers-Danlos syndrome (EDS). EDS is a diverse group of heritable connective tissue disorders, which include 6 major types and additional minor subtypes typically characterized by skin hyperextensibility and hypermobile joints^2^,^17^. Since the pathogenesis of EDS generally involves abnormalities in collagen fibrillogenesis^18^, we investigated whether MOWS patients also show abnormalities in the dermis. Further, we generated mesoderm-specific *Zeb2* conditional knockout (*Zeb2*-cKO) mice, which reproduced the skin phenotype found in MOWS patients.

## Results

### Patients with MOWS exhibit an EDS-like phenotype

**Figure 1:**
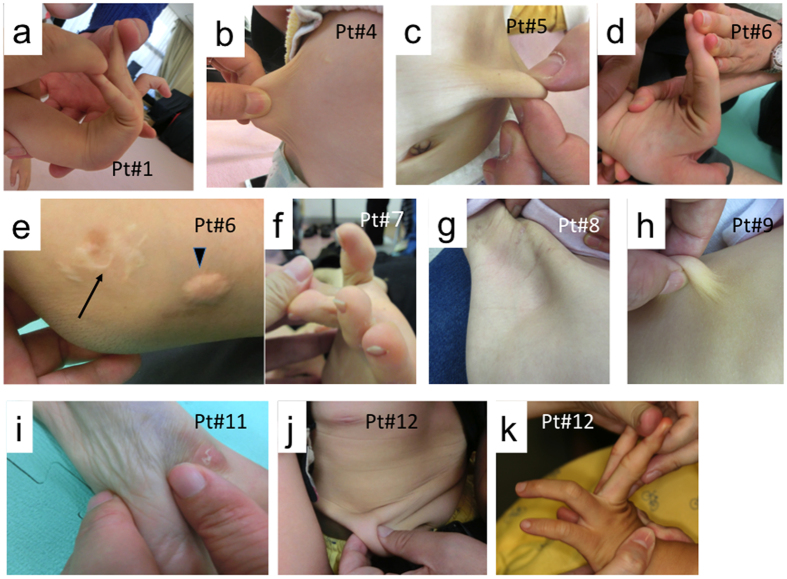
EDS-like appearance in patients with MOWS. (**a,d,f,k**) Joint hypermobility. (**b,c,h,j**) Skin hyperextensibility. (**e**) Atrophic cutaneous scars (arrow), molluscoid pseudotumor of the elbow (arrowhead). (**g,i**) Skin thinning. Patient numbers are shown in each panel (see Supplementary Table 1 for listing).

We enrolled 12 patients with MOWS (2–16 years of age), 8 of whom had been previously reported^3^,^19^ along with diagnoses based on genotype findings in their *ZEB2* gene and/or clinical features. Those features include a characteristic facial appearance, microcephaly, congenital heart disease such as ventricular septal defect, neurological symptoms such as intellectual disability and seizure, Hirschsprung disease, and agenesis or hypoplasia of the corpus callosum (Supplementary Table 1). We recognized that all 12 of those MOWS patients had soft and velvety skin. Furthermore, they exhibited some of the characteristic features of EDS, including skin hyperextensibility (Fig. 1b,c,h,j), joint hypermobility (Fig. 1a,d,f,k), atrophic cutaneous scars and a molluscoid pseudotumor of the elbow (Fig. 1e), which are characterized as classical symptoms of EDS^20^. Joint laxity was evident in the fingers, wrists and toes (Fig. 1a,d,f,k), although their symptoms did not reach a Beighton score of 5 or more, which is the standard assessment of joint disease in EDS^21^. Some patients had thin, almost translucent skin and therefore their veins were easily visible (Fig. 1g,i). However, they did not develop easy bruising as do patients with vascular EDS. It should be noted that the skin phenotypes found in MOWS patients did not correlate with *ZEB2* mutations or the severity of their clinical features (Supplementary Table 1). Ultrasonic examination of two representative patients (patients #11 and #12) revealed that the dermal thickness in various parts of their body was generally thinner than in age-matched healthy controls (Supplementary Fig. 1).

### Ultrastructural characteristics of the dermis of a MOWS patient

**Figure 2:**
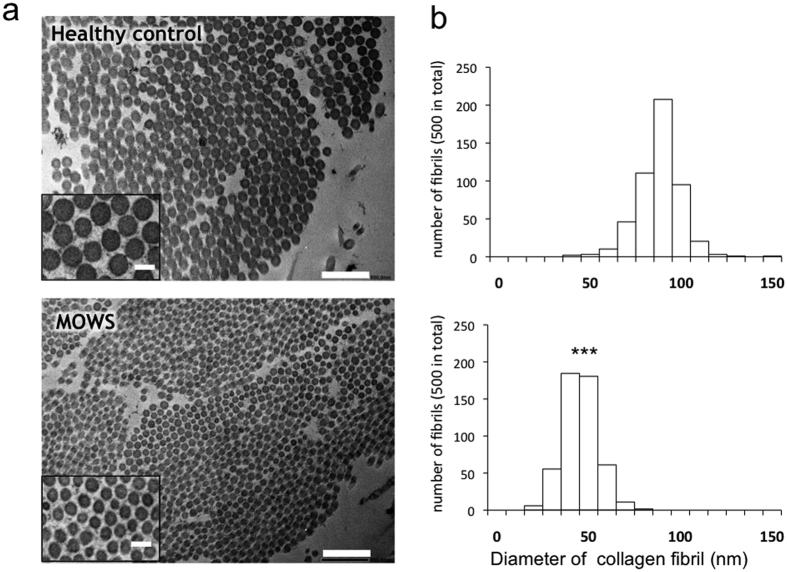
Miniaturized collagen fibrils in the dermis of a MOWS patient. (**a**) Electron micrographs of the dermis of a healthy control (top) and a MOWS patient (#11, bottom). Scale bars, 500 nm, 100 nm (insets). (**b**) Diameters of collagen fibrils in the healthy control (top) and in the MOWS patient (bottom). Diameters of 500 fibrils were counted and are shown in 10 nm increments. ***P < 0.001 by Student’s t-test.

Electron microscopic examination of a skin biopsy from a representative MOWS patient (patient #11) revealed marked miniaturization in the diameter of collagen fibrils compared with a healthy control (Fig. 2; mean nm ± SD; 92.96 ± 11.94 in the healthy control, 50.6 ± 9.32 in MOWS; *P *< 0.001 by Student’s t-test). Similarly, the dermis of EDS patients shows abnormalities in collagen fibrils by electron microscopy, including heterogeneity in size with a flower-like shape^22^. Although the features were not identical, the ultrastructural abnormality in collagen fibrils strongly suggests that MOWS patients exhibit the EDS-like skin phenotype possibly due to abnormal fibrillogenesis of collagen in the dermis.

### Decreased ZEB2 expression in dermal fibroblasts from a MOWS patient

MOWS is caused by *de novo* heterozygous mutations or deletions in the *ZEB2* gene^2^, which leads to haploinsufficiency^7^. RT-PCR revealed that *ZEB2* gene expression in dermal fibroblasts derived from MOWS patient #11, who had a frameshift mutation (p.T761Kfs_26)^3^, was decreased by approximately 40% compared to healthy controls, whereas the level of *ZEB1* mRNA was not affected (Supplementary Fig. 2a). Correspondingly, the ZEB2 protein level of fibroblasts derived from MOWS patient #11 was reduced close to half of the levels in control fibroblasts (Supplementary Fig. 2b,c). Collectively, these results confirmed the *ZEB2* haploinsufficiency in MOWS patients.

### Generation of *Zeb2*-cKO mice using the Cre-loxP system

To study the role of Zeb2 *in vivo*, we generated Cre-mediated *Zeb2*-cKO mice (Supplementary Fig. 3a), since a germline *Zeb2* deficiency leads to embryonic lethality^13^. To this end, we crossed *Zeb2* floxed mice with mice harboring the *Cre* transgene under the promoter of the *Prx1* gene, which is specific for the mesoderm/mesenchyme^23^,^24^. Prx1-Cre:Zeb2^flox/flox^ (*Zeb2*-cKO) mice were born in accordance with the Mendelian law. Genomic PCR analysis of newborn *Zeb2*-cKO mice revealed that the gene targeting occurred in dermal fibroblasts and in subcutaneous fat tissues, but not in epidermal cells or the liver, indicating that it was mesenchymal-specific (Supplementary Fig. 3b).

### *Zeb2*-cKO mice show a skin phenotype similar to MOWS

**Figure 3:**
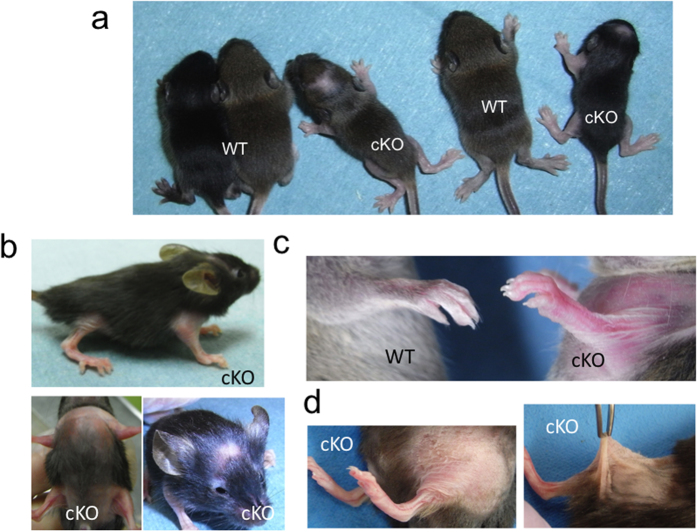
Gross appearance of *Zeb2*-cKO mice. (**a**) Wild-type (WT) and *Zeb2*-cKO (cKO) mice at 2 weeks of age. Growth retardation and local alopecia in the scalp were found in *Zeb2*-cKO mice. (**b**) Alopecia developed in the scalp, limbs, chest and abdomen of *Zeb2*-cKO mice at 4 weeks of age. (**c**) Redundant skin in the upper limb of *Zeb2*-cKO mice (right) at 8 weeks of age. (**d**) *Zeb2*-cKO mice (right) at 16 weeks of age showed redundant skin and hyperextensibility.

**Figure 4:**
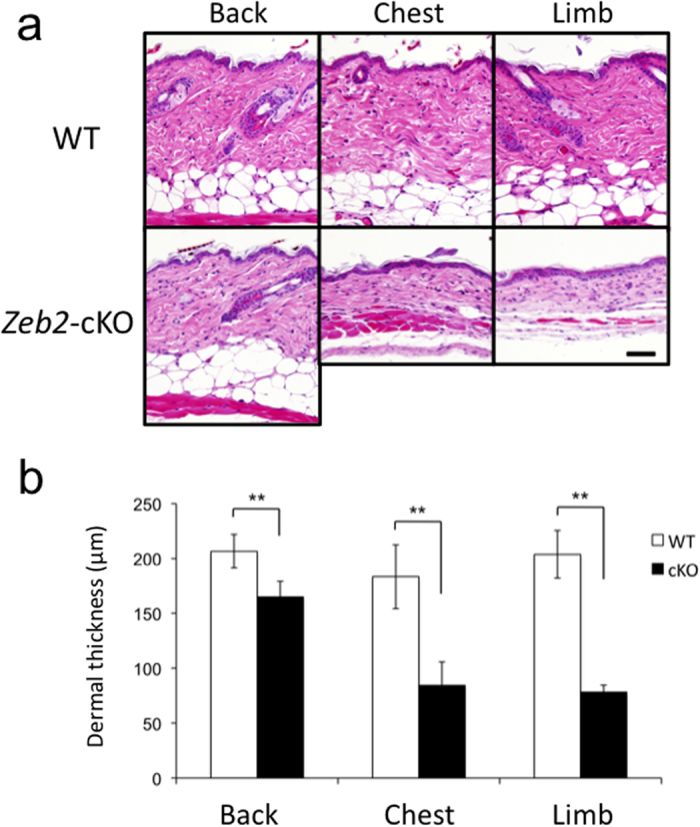
Reduced dermal thickness in *Zeb2*-cKO mice. (**a**) Histological features of the back, chest and limb skins of wild-type mice (WT, top panels) and *Zeb2*-cKO mice (bottom panels) at 7 weeks of age. Scale bar, 50 μm. (**b**) Dermal thickness (μm) of WT (white bars, n = 3) and *Zeb2*-cKO mice (black bars, n = 3). **P < 0.01 by Student’s t-test.

**Figure 5:**
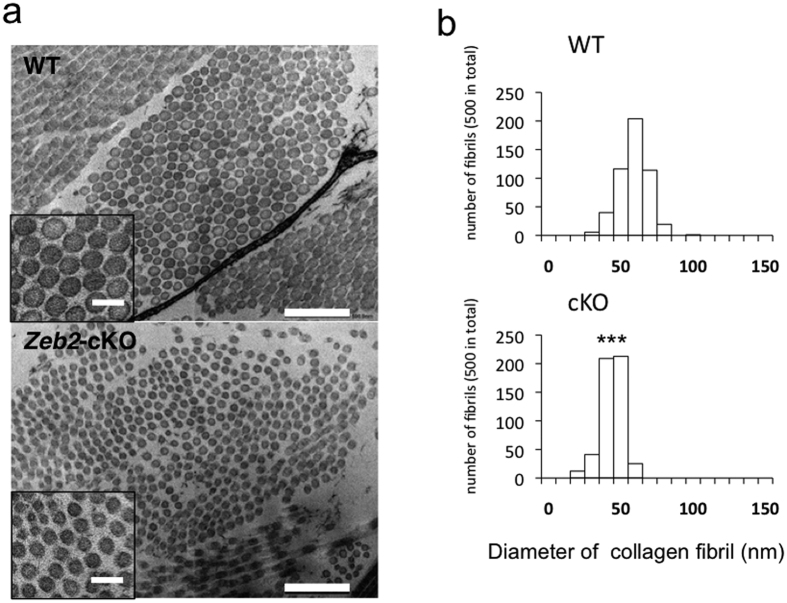
Miniaturized collagen fibrils in the dermis of *Zeb2*-cKO mice at 7 weeks of age. (**a**) Electron micrographs of the dermis of wild-type (WT, top) and *Zeb2*-cKO (bottom) mice at 7 weeks of age. Scale bars, 500 nm, 100 nm (insets). (**b**) Diameters of collagen fibrils in WT (top) and *Zeb2*-cKO (bottom) mice. Diameters of 500 fibrils were counted and are shown in 10 nm increments. ***P < 0.001 by Student’s t-test.

Although the gross appearance of newborn *Zeb2*-cKO mice was normal, from two weeks of age onward they showed some growth retardation and hair loss in the scalp and extremities (Fig. 3a,b). Strikingly, they exhibited redundant, hyperextensive skin, which resembled that found in EDS patients (Fig. 3c,d). Furthermore, histopathology revealed that the dermis of *Zeb2*-cKO mice had a reduced thickness compared with wild-type control mice (Fig. 4a,b). In addition, subcutaneous fat tissues in the chest and limbs, but not in the back, were atrophic compared with controls (Fig. 4a). Electron microscopic analysis of the dermis revealed uniform miniaturization of collagen fibrils in *Zeb2*-cKO mice both at 7 (Fig. 5) and at 14 weeks of age (Supplementary Fig. 4) compared with wild-type mice. Therefore, the ultrastructural characteristics of *Zeb2*-cKO mice were identical to those found in MOWS patients. Collectively, the skin abnormalities of MOWS patients were faithfully reproduced in *Zeb2*-cKO mice, suggesting that *ZEB2* gene disruption might lead to impairment in the homeostasis of the dermal structure such as collagen synthesis.

### Abnormal teeth and craniofacial development in *Zeb2*-cKO mice

In addition to changes in skin phenotypes, *Zeb2*-cKO mice showed abnormalities in the development of their craniofacial bones and teeth (Supplementary Fig. 5a,b). Histological examination of the teeth of *Zeb2*-cKO mice revealed a reduction of alveolar bone volume, cellular cementum hyperplasia and a morphological abnormality in the root apex (Supplementary Fig. 5c). The abnormalities in craniofacial bone and teeth observed in *Zeb2*-cKO mice might be relevant to the characteristic microcephaly and frequently found malalignment of teeth in MOWS patients.

### Abnormalities in gene expression related to collagenogenesis in dermal fibroblasts of *Zeb2*-cKO mice

**Figure 6:**
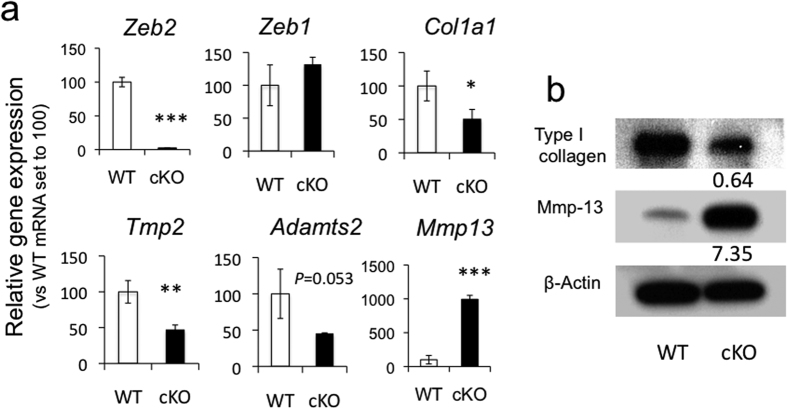
Down-regulation of genes involved in collagenogenesis and up-regulation of genes involved in collagenolysis in dermal fibroblasts derived from *Zeb2*-cKO mice. (**a**) mRNA levels of dermal fibroblasts from *Zeb2*-cKO mice (cKO) relative to wild-type (WT), which are set to 100. *, **, ***P < 0.05, 0.01, 0.001, respectively, by Student’s t-test. n = 3–5. (**b**) Western blot analysis. Numbers indicate relative folds of proteins from *Zeb2*-cKO fibroblasts compared with WT fibroblasts after normalization with β-actin proteins. Bands are displayed as cropped images from original blots on the gel (see Supplementary Information).

To examine the changes in gene expression in dermal fibroblasts derived from *Zeb2*-cKO mice, we performed DNA microarray analysis. Transcripts with at least a 4-fold difference in expression level between *Zeb2*-cKO fibroblasts and wild-type fibroblasts were selected and sorted with ECM-related genes (Supplementary Fig. 6). Many genes encoding collagen, Timps and other proteins known to be involved in connective tissue diseases such as EDS and Marfan syndrome, including *Adamts2, Fbn1* and *Fbn2*, had more than a 4-fold decreased expression in *Zeb2*-cKO fibroblasts compared with wild-type fibroblasts. In contrast, genes encoding Mmps were on the list of genes with increased expression in *Zeb2*-cKO fibroblasts. Real time RT-PCR analysis reproduced, in part, the results of the microarray analysis, such as the down-regulation of *Col1a1, Tmp2* and *Adamts2* and the up-regulation of *Mmp13* (Fig. 6a). Furthermore, Western blot analysis revealed a reduced expression level of Type 1 collagen and a markedly increased expression level of Mmp13 protein (Fig. 6b). Taken collectively, dermal fibroblasts derived from *Zeb2*-cKO mice exhibited changes in ECM-related molecules, which are involved in the down-regulation of collagenogenesis and in the up-regulation of collagenolysis.

### Impairment of bleomycin-induced collagen biosynthesis in *Zeb2*-cKO mice

**Figure 7:**
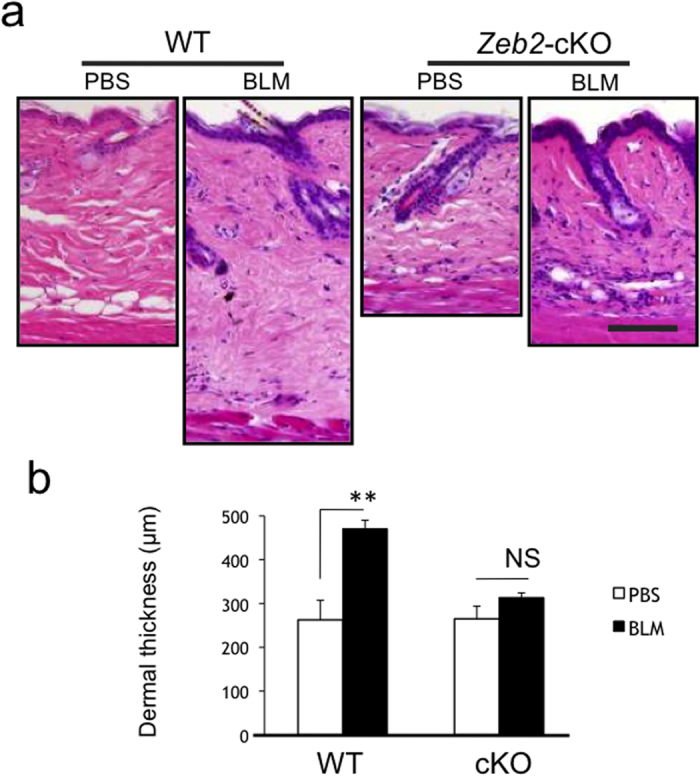
Attenuated bleomycin-induced dermal fibrogenesis in *Zeb2*-cKO mice. (**a**) Histological views of the dermis in 12-week-old wild-type (WT) and *Zeb2*-cKO mice after treatment with PBS or bleomycin (BLM) (H&E). Scale bar, 100 μm. (**b**) Mean dermal thickness (μm) ± SD in WT mice (n = 4) and in *Zeb2*-cKO mice (n = 3). **P < 0.01; NS, not significant, by Student’s t-test.

Finally, we investigated whether the *Zeb2* deletion affected inducible skin fibrosis. Bleomycin was subcutaneously injected every other day in the backs of 6 to 8-week-old mice for 4 weeks. Strikingly, the fibrogenic response of the dermis to bleomycin was greatly attenuated in *Zeb2*-cKO mice compared with wild-type mice (Fig. 7). This result indicated that Zeb2 is required not only for constitutive fibrogenesis but also for the inducible fibrogenesis of the skin.

## Discussion

ZEB2 has been recognized to be a multifunctional regulator of nervous system development^25^, since mutations in the *ZEB2* gene lead to severe neurological consequences in animal models^12^,^13^,^14^,^26^, which recapitulates a number of symptoms of MOWS patients. Heterozygous *Zeb2* knockout mice display decreased thermal pain responses, suggesting that Zeb2 contributes to thermal pain sensitivity via coordinated changes in DRG-neuron voltage-gated ion channels^27^. To circumvent embryonic lethality and/or underscore the role for ZEB2 in detail, homozygous deletion of Zeb2 has been conducted through the Cre-loxP system under organ-specific promoters. Neural crest-specific *Zeb2* knockout mice display craniofacial and gastrointestinal malformations^14^. *Zeb*2 deletion specifically in the cerebral cortex affects development of the hippocampus and dentate gyrus^28^. Further, conditional deletion of Zeb2 in postmitotic neurons results in premature generation of layer 2–5 neurons and defects in ipsilateral neocortical axonal growth^29^,^30^. A recent study demonstrated that *de novo* heterozygous *Zeb2* KO mice, which were established by inducing the *Zeb2* mutation in germ cells, develop multiple defects relevant to MOWS, including craniofacial abnormalities and defective corpus callosum formation^26^. However, no description was made as to whether they exhibit a skin phenotype.

Indeed, there has been a paucity of literature on the skin phenotypes in patients with MOWS, but only brief descriptions of a fair complexion^15^ or raindrop depigmentation have been made^16^. In this study, we report for the first time that most patients with MOWS have skin symptoms similar to EDS patients, which are characterized by skin hyperextensibility, thin, translucent skin with visible veins, atrophic scarring and joint hypermobility. While the typical electron microscopy findings of the dermis in EDS are called ‘collagen flowers’ or ‘cauliflowers’^20^, those in patients with MOWS are miniaturized fibrils, indicating a distinct difference from EDS but a common mechanism of abnormal fibrillogenesis in the dermis. A ZEB2 deficiency should be the underlying cause of that abnormality, since the same change was found in *Zeb2*-cKO mice.

To explain this hypothesis, we generated *Zeb2*-cKO mice under control of the Prx1 promoter, which is expressed throughout the early limb bud mesenchyme and in a subset of the craniofacial mesenchyme^24^. Indeed, Cre-mediated recombination occurred in dermal fibroblasts and in subcutaneous fat tissues, but not in epidermal keratinocytes or liver tissues. Abnormal craniofacial bone and teeth development in *Zeb2*-cKO mice might be attributed to the *Zeb2* deletion in the craniofacial/teeth mesenchyme and mimic, in part, the characteristic facial and dental features of MOWS patients, including hypertelorism, medially flared and broad eyebrows, a prominent columella, pointed chin, uplifted earlobes and malpositioned teeth^5^,^15^.

Although patients with MOWS have heterozygous mutations in the *ZEB2* gene, mice having a heterozygous *Zeb2* gene deletion in the present study (Prx1-Cre:Zeb2^flox/wild^) showed no symptoms in the skin, craniofacial or teeth development. The discordance with this mouse model might be attributed to the nature of the Prx1 promoter for Cre recombinase. The reduction of the dermal thickness and the occurrence of alopecia somewhat depended on the sites of *Zeb2*-cKO mice; that is, it was more pronounced on the limbs, scalp and interlimb flanks, but less on the back. This could be due to site-specific differences in expression of the *Prx1* enhancer^24^.

The most striking feature of *Zeb2*-cKO mice was their skin phenotype, including a redundant, hyperextensible thin skin, which resembled, in part, the skin phenotype found in MOWS patients that display EDS-like symptoms. EDS is a heterogeneous connective tissue disorder through various pathomechanisms consisting of dominant-negative effects of mutant or haploinsufficiency of procollagen α-chains, and deficiency of collagen-processing enzymes^18^. *Zeb2*-cKO mice display a redundant, thin dermis with histological and ultrastructural changes and an attenuated dermal fibrogenesis by bleomycin treatment, which represents an EDS-like abnormality in the homeostasis of the dermis. However, it remains undefined so far whether *Zeb2*-cKO mice have joint hypermobility or vascular changes, as found in classical EDS. Together with the results from experiments using *Zeb2*-cKO mice, we speculate that the EDS-like skin phenotype in MOWS patients might result from abnormal collagen biosynthesis by dermal fibroblasts due to *ZEB2* mutations or deletions. However, *Zeb2*-cKO mice display hair loss (alopecia), which MOWS and EDS patients do not develop.

ZEB2, previously termed Smad interacting protein 1 (SIP1), acts as a transcriptional co-repressor in the TGF-β signaling pathway^8^,^11^, but it can also act as a transcriptional activator^31^. ZEB2 also interacts with nucleosome remodeling and the histone deacetylation (NuRD) complex^32^. Through these molecular interactions, ZEB2 plays a critical role in neural crest cell migration, and therefore, embryos harboring homozygous deletions of the Zeb2 gene exhibit compromised development of the peripheral nervous system, craniofacial tissue and heart^12^,^13^. We did not find any impairment in *in vitro* cell proliferation, invasive or migratory activity of fibroblasts of *Zeb2*-cKO mice (unpublished observations). In contrast, fibroblasts derived from Zeb2-deficient mice show altered expression profiles of genes that would increase collagenolysis but would reduce collagenogenesis. Some of the down-regulated genes identified in the DNA array analysis involve those responsible for hereditary connective tissue disorders, including *collagens* and *ADAMTS2* for EDS and *fibrillin-1* for Marfan syndrome. Further studies will be necessary to investigate how ZEB2, as a transcriptional regulator, mechanistically contributes to ECM homeostasis such as collagen fibrillogenesis in the skin. In conclusion, we demonstrate for the first time that mesoderm-specific deletion of *Zeb2* leads to the abnormality in the dermis of an EDS-like skin phenotype, which was also observed in patients with MOWS. In addition, ZEB2 would be a novel therapeutic target for pathogenic diseases involving fibrosis, including liver cirrhosis, cardiac fibrosis, pulmonary fibrosis and scleroderma.

## Methods

### Patients

Twelve patients, who had been diagnosed with MOWS based on clinical symptoms and mutation or deletion analysis of the *ZEB2* gene, were inspected in detail for their skin appearance by 3 dermatologists (M.T., S.S., N.K.). Informed consent was obtained from all patients whose information and images are presented in this study. Skin biopsies were taken from patients #11 and 12 after obtaining written informed consent. The experiments were conducted at the Department of Dermatology, Kochi Medical School Hospital after approval by the Institutional Review Board at Kochi University in accordance with the Declaration of Helsinki Principles.

### Ultrasound measurement of skin thickness

Patients and age-matched healthy controls were measured with an ACUCON P300 ultrasound system using a LA435 convex transducer (Siemens Healthineers, Tokyo, Japan), to measure skin thickness at different sites of the body, including the arm, breast, abdomen, back, lumbar, femoral and popliteal areas. Dermal thickness was measured on the B-mode image.

### Electron microscopic examination

Skin specimens were fixed in 2.5% glutaraldehyde for 2 h, post-fixed in 1% osmium tetroxide for 2 h, dehydrated in a graded ethanol series, and embedded in epoxy resin. Semithin sections (1 μm) were stained with toluidine blue. Ultrathin sections (100 nm) were stained with uranyl acetate and lead citrate and were examined with a JEM-1400Plus transmission electron microscope (JEOL Ltd. Tokyo, Japan).

### Primary fibroblast culture

The dermis of biopsied skin specimens after removal of the epidermis using dispase (BD Biosciences, Tokyo, Japan) was cut into pieces with a scalpel, then soaked in Dulbecco’s modified Eagle’s medium with 10% heat-inactivated fetal bovine serum, 100 U/mL penicillin, 100 U/mL streptomycin and 2 mM L-glutamine (Invitrogen, Carlsbad, CA). Five to 7 days later, propagated fibroblasts were collected and split. Cells were used for experiments at the 2^nd^ to 4^th^ passages.

### Generation of Prx1-Cre:Zeb2^flox/flox^ mice

Mice that carried the *Cre* recombinase gene under control of the mesoderm-specific regulatory element Prx1^24^ were crossed with mice that were homozygous for the *Zeb2* floxed allele^12^ to generate Prx1-Cre/Zeb2^**flox/0**^ mice. The second cross with Zeb2^**flox/flox**^mice resulted in the generation of Prx1-Cre:Zeb2^flox/flox^ mice. Allele-specific PCR was carried out as illustrated in Supplementary Fig. 3a. The primers were designed to detect the *Zeb2* floxed allele using primers P1 and P2 (339 bp), and the truncated allele using P1 and P3 primers (288 bp). The DNA sequences of primers 1, 2 and 3 are as follows. P1,

5′-GAACTAGTTGAATTGGTAGAATCAATGGGG-3′, a sense sequence of intron 6; P2, 5′-GTAAAGGCTCTCTACGCCTTTTTCAGTTAG-3′; an antisense sequence of intron 6; P3, 5′-AAGCATGTCGGTAAGCTGACCAACTACTAG-3′, an antisense sequence of intron 7. Using a mixture of these primers, PCR was performed with 35 cycles of a reaction consisting of 30 sec of denaturation at 94 °C, 30 sec of annealing at 50 °C and 1 min of elongation at 72 °C.

### DNA microarray analysis

Total RNAs were extracted from cells using the TRIzol reagent according to the manufacturer’s instructions (Life Technologies, Carlsbad, CA). The DNA microarray analysis was performed using Affymetrix GeneChip Mouse 430 2.0 Arrays according to the manufacturers’ instructions. Image files were scanned and processed by AGCC (Affymetrix GeneChip Command Console Software, Santa Clara, CA) and the microarray data were normalized with GCRMA. Transcripts with at least a 4-fold change difference in expression level between *Zeb2*-cKO and control samples were selected and used for further analysis. A gene ontology (GO) enrichment analysis^33^ was performed via a tool implemented in the RefDIC web server^34^ (http:// refdic.rcai.riken.jp/). A GO term with a corrected p-value < 0.05 was considered as enriched in the selected transcripts. The raw data for the microarray data are available from the RefDIC web server under the accession numbers RSM01584 and RSM01585.

### Quantitative RT-PCR

Total RNAs were extracted using an RNA isolation kit (Promega, Madison, WI) according to the manufacturer’s protocol, and were reverse-transcribed using M-MLV reverse transcriptase (Invitrogen) with random oligonucleotide hexamers (Invitrogen). PCR reactions were performed using *Power* SYBR Green PCR Master Mix (Applied Biosystems, Foster City, CA), and amplification conditions were as follows: 50 °C for 2 min, 90 °C for 10 min for 1 cycle, followed by 40 cycles of 95 °C for 15 s and 60 °C for 1 min. The primers used were as follows (sense and antisense, respectively); mouse *Zeb1*, 5′-GCCAGCAGTCATGATGAAAA-3′ and 5′-TATCACAATACGGGCAGGTG-3′; mouse *Zeb2,* 5′-ACCAAATGCTAACCCAAGGA-3′ and 5′-GGCATTCGTAAGGTTTTTCA-3′; mouse *Timp2*, 5′-AGTGATTTCCCCGCCAACTC-3′ and 5′-AAGGGGGCCATCATGGTATC-3′; mouse *Adamts2*, 5′-CGTGGAGTGGCAGGGTGAG-3′ and 5′-CCTCCATCCGGATCAGACCA-3′; *Mmp13*, 5′-GCCAGAACTTCCCAACCAT-3′ and 5′-TCAGAGCCCAGAATTTTCTCC-3′; mouse HPRT, 5′-TGACCTTGATTTATTTTGCATACC-3′ and 5′-CGAGCAAGACGTTCAGTCCT-3′. The quantity of each transcript was analyzed using the 7300 Fast System Software (Applied Biosystems) and was normalized to hypoxanthine phosphoribosyltransferase (HPRT) according to the ∆∆Ct method.

### Western blot analysis

Cell lysates were prepared using RIPA buffer (Sigma-Aldrich, Tokyo, Japan), separated on 4–15% gradient gels (Bio-Rad, Tokyo, Japan) and blotted on polyvinyl difluoride (PVDF) membranes (Bio-Rad). The membranes were incubated with antibodies including; anti-ZEB2 Ab (Santa Cruz Biotechnology, Dallas, TX), anti-HPRT Ab (GeneTex, Irvine, CA), anti-mouse type I collagen (CedarLane Laboratories, Burlington, Canada), anti-MMP13 Ab (Abcam, Cambridge, United Kingdom) and anti-beta actin Ab (Sigma-Aldrich), followed by HRP-conjugated secondary antibodies of relevant species (Cell Signaling, Tokyo, Japan). An ECL Prime kit (GE Healthcare, Tokyo, Japan) was used for signal detection. ImageJ (NIH) was used for quantification of the signals.

### Bleomycin-induced fibrogenesis assay

Bleomycin hydrochloride (Wako Pure Chemical Industries, Ltd., Osaka, Japan) was dissolved in phosphate-buffered saline (PBS) at 500 μg/mL and sterilized with filtration. One hundred μl BLM (50 μg) or PBS were injected subcutaneously into the shaved backs of *Zeb2*-cKO mice or wild-type mice at 8 weeks of age every other day for 4 weeks. The injected skin was removed and processed for histological analysis.

### Statistical analysis

Samples were compared with two-tailed, unpaired Student’s *t*-test. *P*-values less than 0.05 are considered significant.

## Additional Information

**How to cite this article**: Teraishi, M. *et al*. Critical involvement of ZEB2 in collagen fibrillogenesis: the molecular similarity between Mowat-Wilson syndrome and Ehlers-Danlos syndrome. *Sci. Rep.* **7**, 46565; doi: 10.1038/srep46565 (2017).

**Publisher's note:** Springer Nature remains neutral with regard to jurisdictional claims in published maps and institutional affiliations.

## Supplementary Material

Supplementary Information
