# Supplementary material for: Critical involvement of ZEB2 in collagen fibrillogenesis: the molecular similarity between Mowat-Wilson syndrome and Ehlers-Danlos syndrome
Source: Sci Rep. 2017 Apr 19;7:46565. doi: 10.1038/srep46565 (PMC5396187; doi:10.1038/srep46565)
Supplement: Supplementary Information [file srep46565-s1.pdf]

Supplementary Information

Critical involvement of ZEB2 in collagen fibrillogenesis: the molecular similarity between Mowat-Wilson syndrome and Ehlers-Danlos syndrome

Mika Teraishi [1], Mikiro Takaishi [1], Kimiko Nakajima [1], Mitsunori Ikeda [1], Yujiro Higashi [2], Shinji Shimoda [3], Yoshinobu Asada [4], Atsushi Hijikata [5], Osamu Ohara [6], Yoko Hiraki [7], Seiji Mizuno [8], Toshiyuki Fukada [9], Takahisa Furukawa [10], Nobuaki Wakamatsu [11], and Shigetoshi Sano [1,*]

[1]Department of Dermatology, Kochi Medical School, Nankoku 783-8505, Japan; [2]Department of Perinatology, Institute for Developmental Research, Aichi Human Service Center, Kasugai 480-0392, Japan; [3]Department of Anatomy, Tsurumi University School of Dental Medicine, Yokohama 230-8501, Japan; [4]Department of Pediatric Dentistry, Tsurumi University School of Dental Medicine, Yokohama 230-8501, Japan; [5]Department of Bioscience, Nagahama Institute of Bio-Science and Technology, Nagahama 526-0829, Japan; [6]Laboratory for Integrative Genomics, RIKEN IMS, Yokohama 230-0045, Japan; [7]Hiroshima Municipal Center for Child Health and Development, Hiroshima 732-0052, Japan; [8]Department of Pediatrics, Central Hospital, Aichi Human Service Center, Kasugai 480-0392, Japan; [9]Department of Molecular and Cellular Physiology, Faculty of Pharmaceutical Science, Tokushima Bunri University, Tokushima 770-8514, Japan; [10]Laboratory for Molecular and Developmental Biology, Institute for Protein Research, Osaka University, Suita 565-0871, Japan; [11]Department of Genetics, Institute for Developmental Research, Aichi Human Service Center, Kasugai 480-0392, Japan

Supplementary Table 1. The profiles of MOWS patients in this study

			ZEB2		characteristic features of MOWS							EDS-like		
Pt #	Age/Sex	ref (pt#)	mut (fs, nsn)	del	ID	CFA	MC	CHD	S	HSCR	ACC/HCC	SH	JH	AS
1	5F		−	NA	+	+	+	+(ASD,VSD)	−	−	HCC	++	++	+
2	13F	19(S19)		*	+	+	+	−	+	+	nd	+	+	−
3	5M		−	NA	+	+	+	+(VSD)	+	+	HCC	+	−	+
4	3M	3(S-131)	c.862_863delG : p.G288Afs*10		+	+	+	−	+	+	ACC	++	−	−
5	2M		1027C>T p.R343X		+	+	+	−	+	−	HCC	++	+	−
6	16F	3(S-97)	c.2083C>T p.R695X		+	+	−	−	+	−	−	+	++	++
7	13M	3(S-32)	c.2083C>T p.R695X		+	+	−	+(VSD)	+	+	nd	+	+	+
8	8F	3(S-90)	c.1417delA p.R473Gfs*14		+	+	+	−	−	+	nd	+	+	+
9	2F		−	NA	+	+	+	−	−	+	ACC	+	+	++
10	11M	3(S-98)	c.2399C>G p.S800X		+	+	+	+(VSD)	+	−	HCC	−	−	+
11	12F	3(S-98)	c.2282delC p.T761Kfs*26		+	+	−	+(PDA)	+	−	ACC	++	+	++
12	10M	3(K-02)	1027C>T p.R343X		+	+	−	−	+	−	−	+	++	+

Pt, patient; mut, mutation; del, deletion; fs, frameshift; nsn, nonsense; ref, reference; NA, not analyzed; *, 1.96 Mb deletion including exons 1 and 2; ID, intellectual disability; CFA, characteristic facial appearance; MC, microcephaly; CHD, congenital heart disease; ASD, atrial septal defect; VSD, ventricular septal defect; PDA, patent ductal arteriosus; S, seizure; HSCR, Hirschsprung disease; ACC, agenesis of the corpus callosum; HCC, hypoplasia of the corpus callosum; nd, not detemined; SH, skin hyperextensibility; JH, joint hypermobility; AS, atrophic scar

a

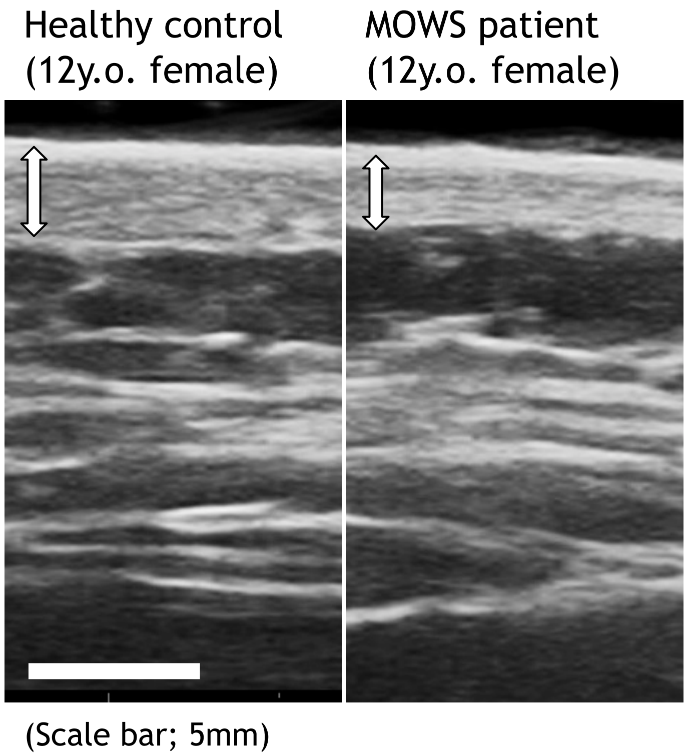

b

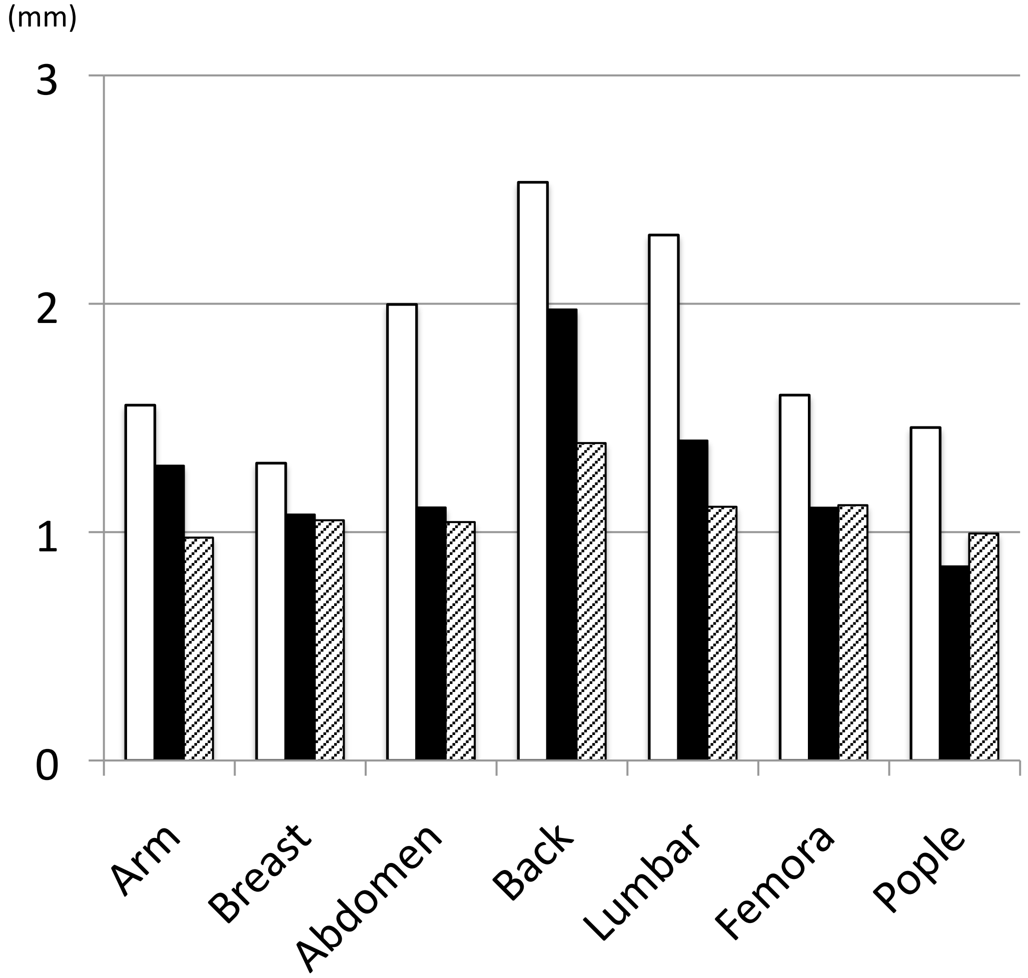

Supplementary Fig. 1

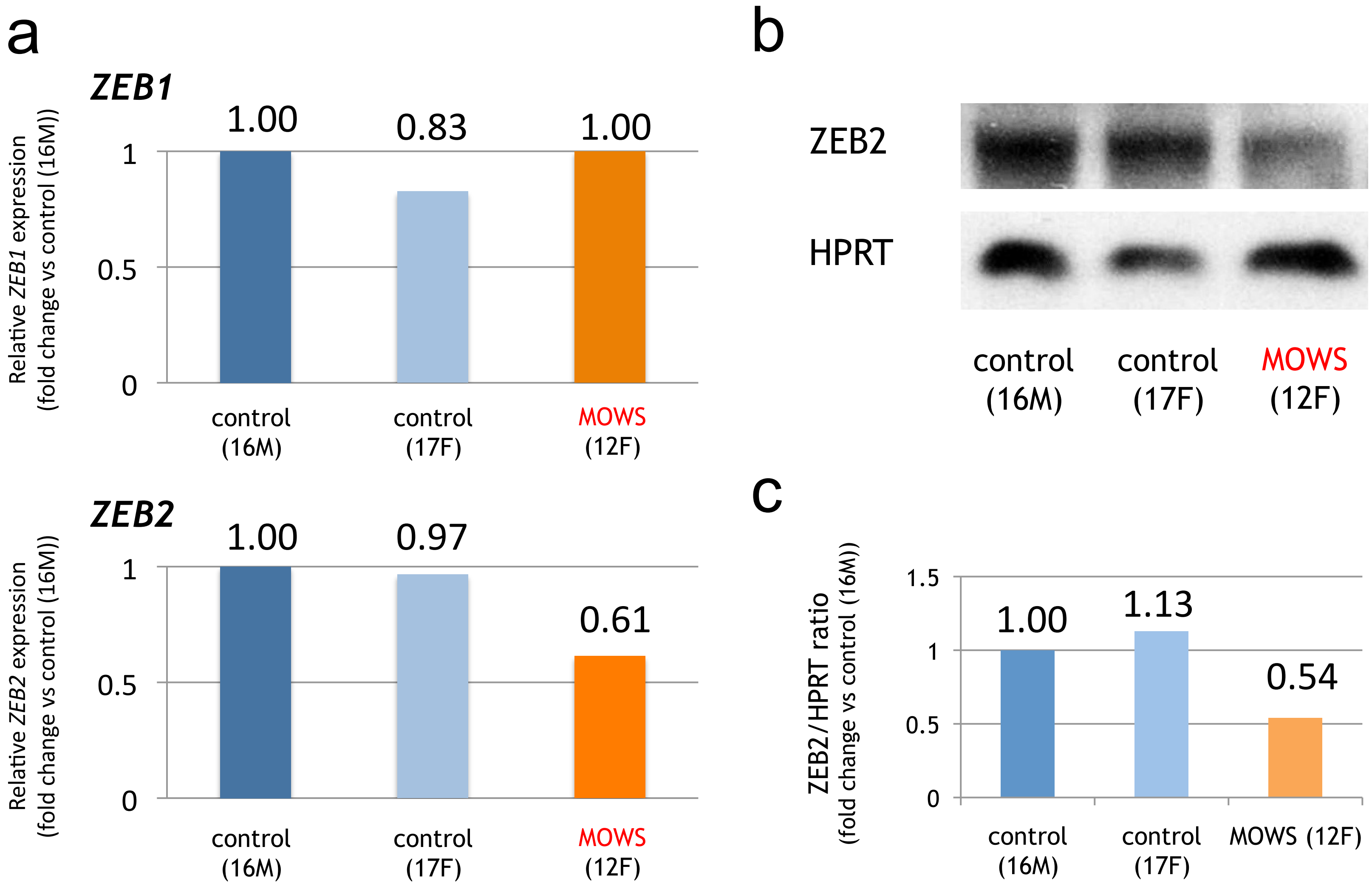

Supplementary Fig. 2

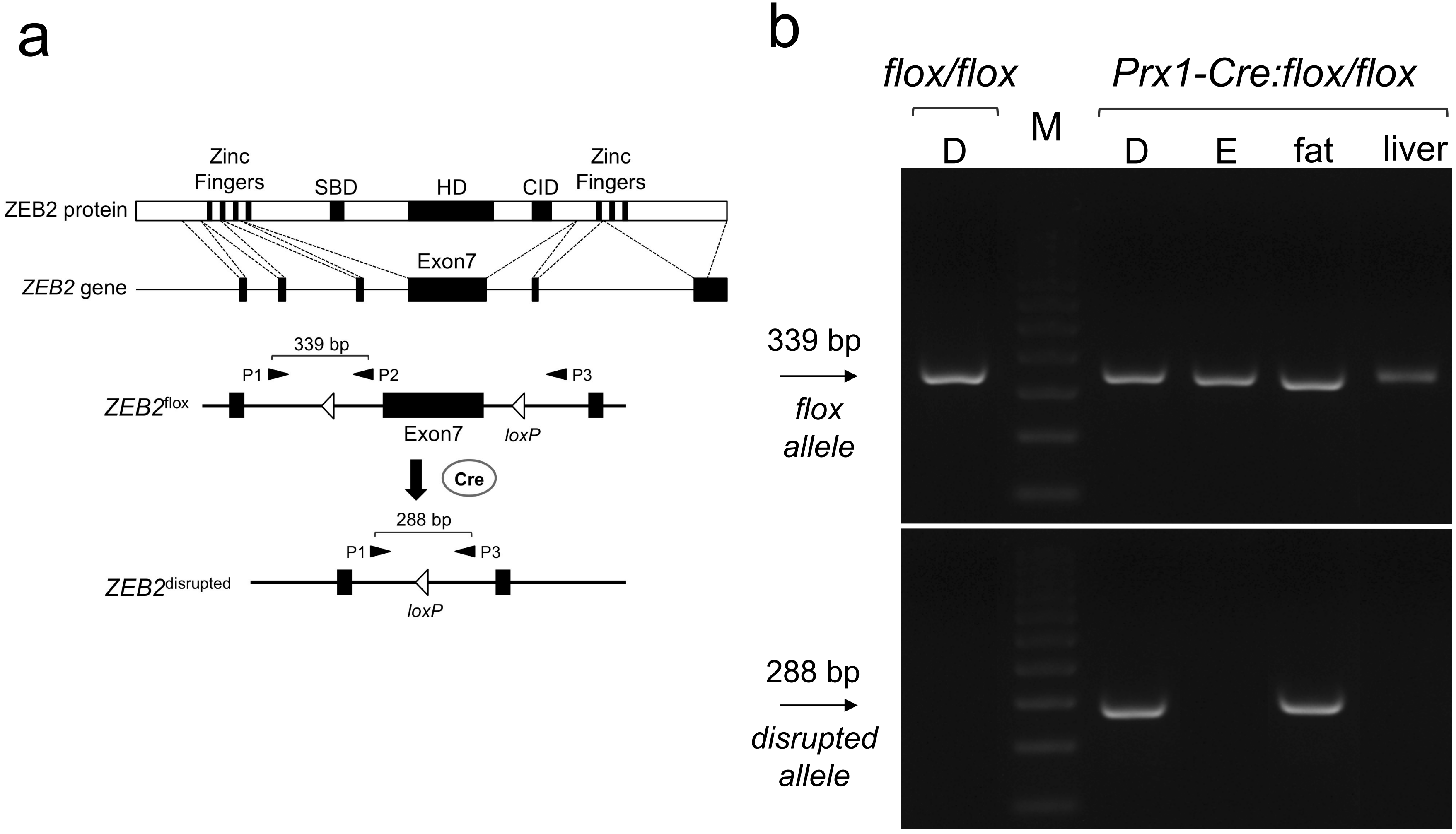

Supplementary Fig. 3

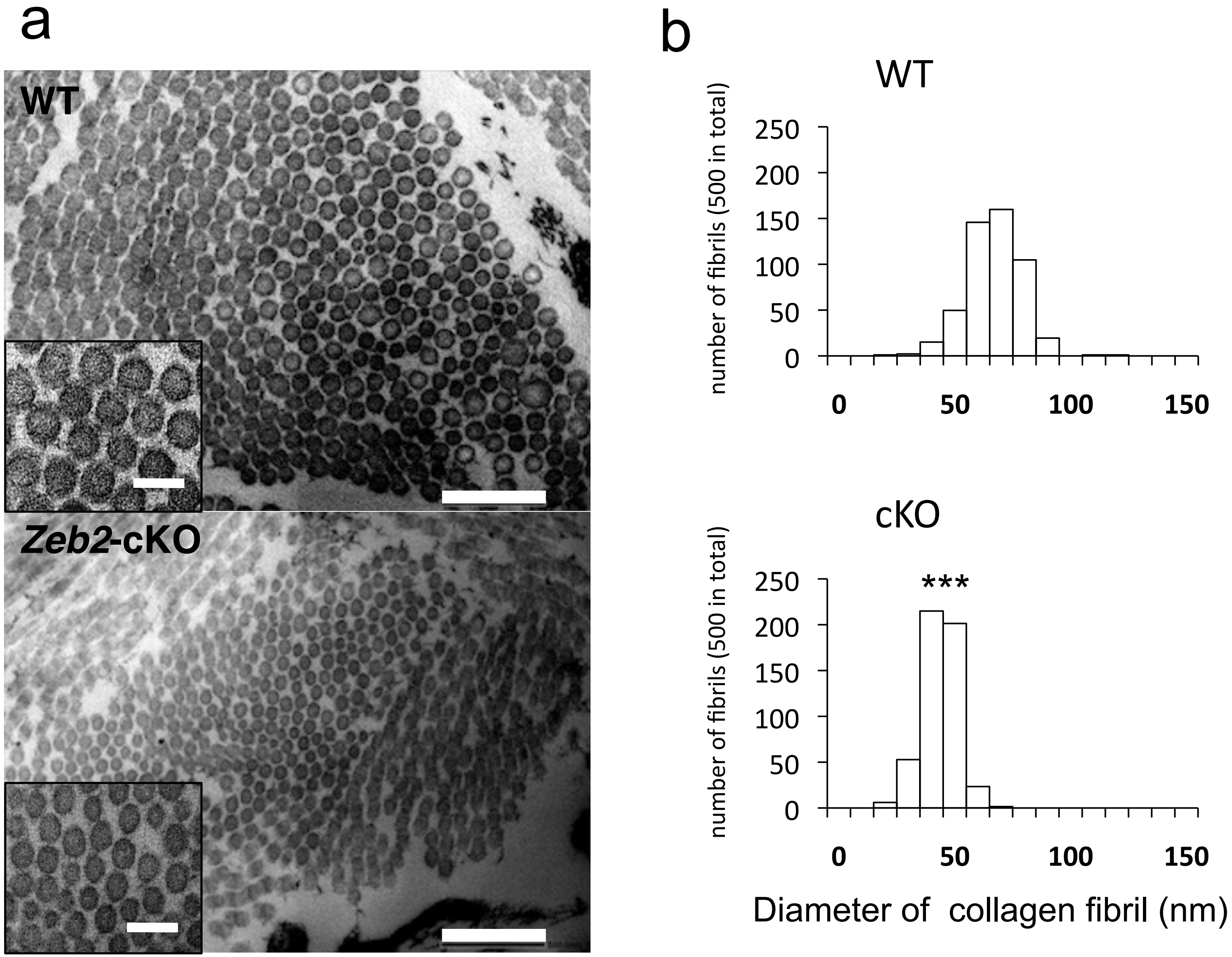

Supplementary Fig. 4

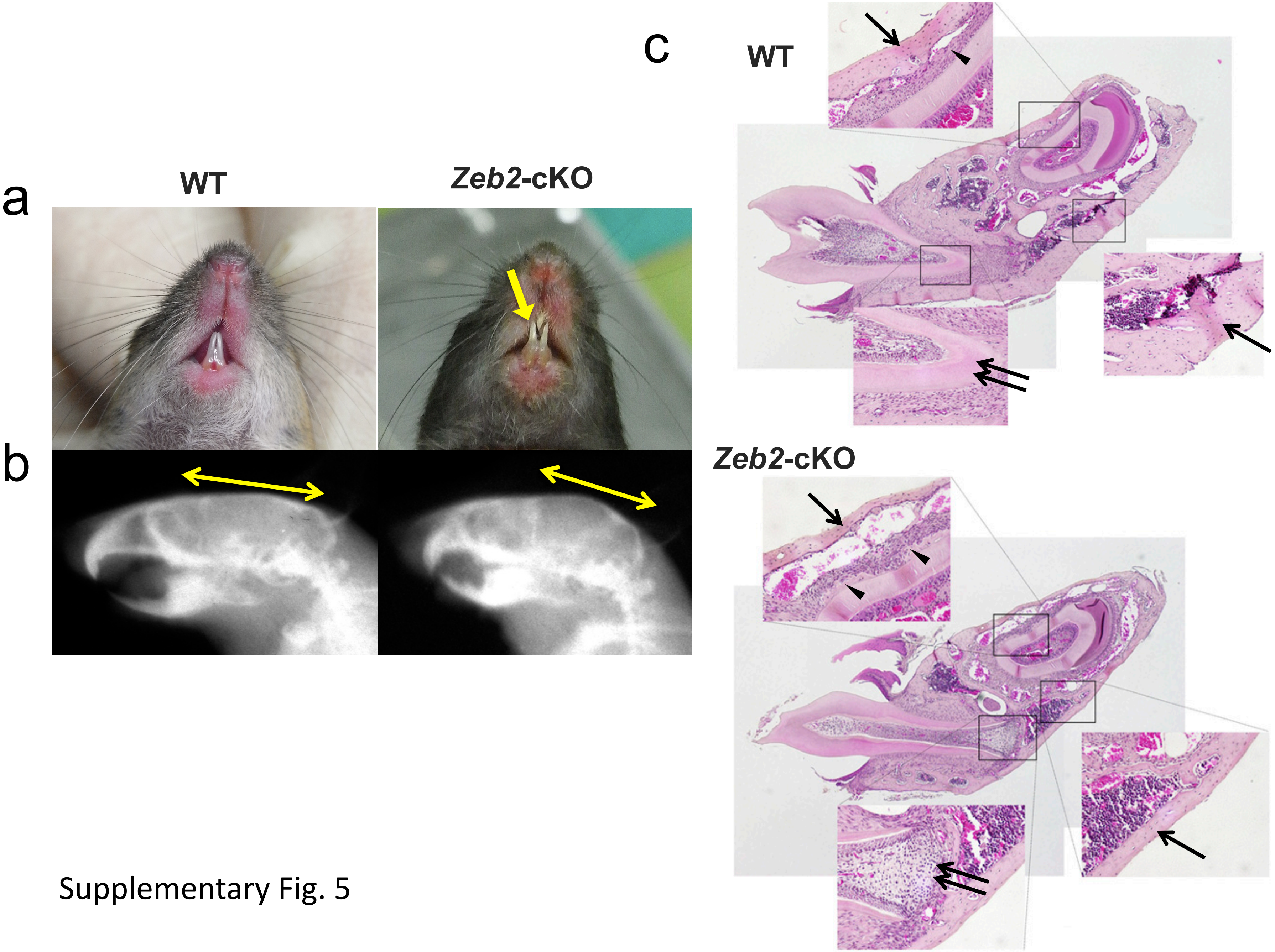

Supplementary Fig. 5

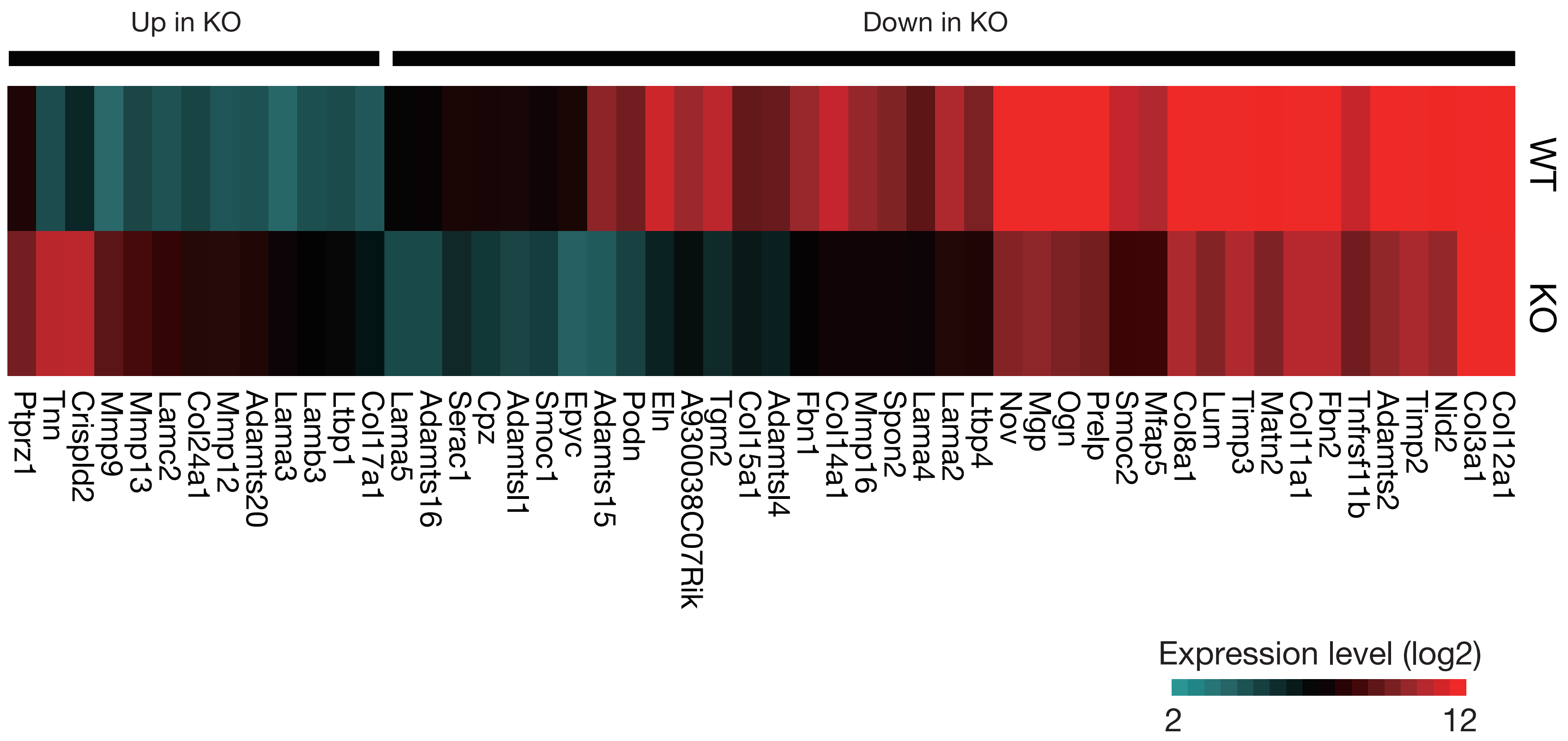

Supplementary Fig. 6

Supplementary Figure 1 Thinning of the dermis in MOWS patients detected by ultrasonography. (a) Comparison of the thickness of the dorsal skin between a MOWS patient (patient #11) and an age-matched healthy control in B-mode scan. Arrow, the dermis. Scale bar, 5 mm. (b) Mean thickness of the dermis in mm at the indicated skin sites. White bars, age-matched healthy controls (n = 6). Black and hatched bars, patients #11 and 12, respectively.

Supplementary Figure 2 (a) Real time RT-PCR analysis for *ZEB1* (upper) and *ZEB2* (bottom) gene expression in cultured dermal fibroblasts derived from a 12-year-old MOWS patient (# 11), who had a frameshift mutation (p.T761Kfs_26), compared with fibroblasts derived from two healthy donors, a 16-year-old boy (16M) and a 17-year-old girl (17F). mRNA levels of *ZEB1* and *ZEB2* were normalized by hypoxanthine-guanine phosphoribosyltransferase (HPRT) mRNA. The primers used were as follows (sense and antisense, respectively); human *ZEB1*, 5'-GTGTACCAGAGGATGACCTGC-3' and 5'-TCCTCCCAGCAGTTCTTAGCA-3'; human *ZEB2,* 5'-CCCTGGCACAACAACGAGA-3' and 5'-GGTCTGGATCGTGGCTTCTG-3'; human HPRT; 5'-TGACCTTGATTTATTTTGCATACC-3' and 5'-CGAGCAAGACGTTCAGTCCT-3'. Arbitrary values of mRNAs are shown relative to the level in fibroblasts of the control (16M), which was set to 1. (b) Western blot analysis of ZEB2 protein levels in cultured fibroblasts derived from the MOWS patient (#11) and healthy controls. (c) Densitometric analysis of the ZEB2 protein. The relative amount of ZEB2 protein in the blot shown in panel (b) was calculated by normalization with HPRT protein. Numbers represent densitometric values.

Supplementary Figure 3 (a) Targeting construct of the Cre-mediated *Zeb2* deletion (Higashi Y*, et al.* (2002) *Genesis* 32(2):82-84). (b) Mesenchymal-specific *Zeb2* deletion. D, dermal fibroblasts; E, epidermal cells; fat, subcutaneous fat tissues; liver, liver tissues; M, molecular markers. Epidermal, dermal cells and subcutaneous fat tissues were taken from the limbs.

Supplementary Figure 4 Miniaturized collagen fibrils in the dermis of *Zeb2*-cKO mice at 14 weeks of age. (a) Electron micrographs of the dermis of wild-type (WT, top) and

Zeb2-cKO mice (bottom). Scale bars, 500 nm, 100 nm (insets). (b) Diameters of collagen fibrils in WT (top) and *Zeb2*-cKO mice (bottom). Diameters of 500 fibrils were counted and are shown in 10 nm increments. ***, $P < 0.001$ by Student's t-test.

Supplementary Figure 5 Abnormal craniofacial and teeth development in *Zeb2*-cKO mice. (a) A 3-week-old *Zeb2*-cKO mouse developed abnormal incisor teeth (arrow). (b) Sagittal images of craniofacial bones by soft X-ray (double-headed arrows) reveal a markedly shortened skull in the *Zeb2*-cKO mouse. (c) Histological views of teeth of wild-type (WT, upper images) and *Zeb2*-cKO (bottom images) mice stained with H&E. Boxed areas are reproduced at a higher magnification. Compared with the tooth of the WT mouse, the *Zeb2*-cKO mouse showed a reduction of alveolar bone volume (arrows), cellular cementum hyperplasia (arrowheads), and a morphological abnormality in the root apex (double arrows).

Supplementary Figure 6 DNA microarray analysis. Transcripts with at least a 4-fold difference in expression level between fibroblasts derived from *Zeb2*-cKO and from wild-type (WT) mice were selected and assorted with ECM-related genes.
